# The reliability and validity of a novel Chinese version simplified modified Rankin scale questionnaire (2011)

**DOI:** 10.1186/s12883-020-01708-1

**Published:** 2020-04-08

**Authors:** Junliang Yuan, Yunxiao Wang, Wenli Hu, Askiel Bruno

**Affiliations:** 1 Department of Neurology, Beijing Chaoyang Hospital, Capital Medical University, Gongti South 8th, Chaoyang, Beijing, 100020 China; 2 Peking University Sixth Hospital, Peking University Institute of Mental Health, NHC Key Laboratory of Mental Health (Peking University), National Clinical Research Center for Mental Disorders (Peking University Sixth Hospital), Beijing, 100191 China; 3 Department of Neurology, Beijing Shunyi Hospital, Beijing, 101300 China; 4 Department of Neurology, Medical College of Georgia, Augusta University, 1120 15th Street, Augusta, GA 30912 USA

**Keywords:** Modified Rankin scale, Simplified modified Rankin scale questionnaire, Stroke, China

## Abstract

**Background:**

The modified Rankin Scale (mRS) is a key global outcome measure after stroke internationally. The latest English version of the simplified modified Rankin scale questionnaire (smRSq)(2011) is a reliable and valid tool in scoring the mRS after stroke. In order to use this tool in Chinese patients, we translated it into Chinese and tested its clinimetric properties.

**Methods:**

The English version smRSq (2011) was translated into Chinese by a standard process. We recruited 300 consecutive hospitalized ischemic stroke patients in the department of neurology, Beijing Chaoyang Hospital. Six randomly paired raters scored the conventional mRS, the novel Chinese version smRSq (2011), the National Institutes of Health Stroke Scale (NIHSS), and the Barthel index (BI) in-person. Inter-rater reliability and validity were assessed.

**Results:**

Among the 300 ischemic stroke patients, mean age was 64.9 ± 12.1 years, and 220 (73%) were male. For inter-rater reliability of the smRSq (2011), the percent agreement among the paired raters was 87%, the kappa (κ) was 0.84 (95% CI, 0.79–0.88), and the weighted kappa (κ_w_) was 0.96 (95% CI, 0.95–0.98). The percent agreement between the smRSq (2011) scores and the conventional mRS scores was 55%, κ = 0.47 (95% CI, 0.40–0.54), and κ_w_ = 0.91 (95% CI, 0.89–0.93). In construct validity testing, the Spearman’s correlation coefficients comparing the smRSq (2011) scores with the NIHSS and the BI scores were 0.83 (*P* < 0.001) and − 0.86 (*P* < 0.001), respectively.

**Conclusions:**

Our results show good to excellent clinimetric properties of the novel Chinese version smRSq (2011) in scoring the mRS in Chinese stroke patients. Further validation in other clinical settings, including in communities and by remote methods in China is warranted.

## Background

China comprises nearly one fifth of the world’s population and the age-standardized prevalence of stroke among Chinese adults has been estimated at 1.1–2.1% (approximately 16–27 million people) [1, 2]. However, the age-specific stroke prevalence increases sharply after the age of 50 years to approximately 5.0–6.7% among people aged 70–79 years.

Assessing functional status after stroke accurately and reliably is a critical part of clinical research and stroke registries. The modified Rankin Scale (mRS) has emerged as the most commonly utilized scale for assessing functional status after stroke [3]. However, because scoring the mRS involves collecting various patient performance data by interviewing patients and caregivers, some subjectivity is inherent. Consequently, its reliability has been measured as suboptimal [4].

Multiple standardized mRS scoring aids have been proposed to improve its reliability [5–9]. These aids consist of prespecified questions and an algorithm to determine the mRS score. One of the simplest, shortest, and validated mRS scoring aids is the latest simplified modified Rankin Scale questionnaire (smRSq)(2011) [8] (Figure S1). This tool has been validated among a wide variety of raters, including over the telephone, with an average time to complete of < 2 min.

We previously translated into Chinese and validated the original smRSq (2010) [10]. Subsequently, the smRSq (2011) showed improved agreements between raters over the original smRSq (2010) [8]. A panel of experts for the International Consortium of Health Outcomes has recommended the smRSq (2011) for standardized mRS scoring [11]. Thus, in this study our aim was to validate a novel Chinese version smRSq (2011). A validated Chinese version smRSq (2011) could facilitate the collection of internationally standardized functional outcome data in Chinese stroke patients. More accurate and standardized data could lead to a better understanding of stroke prognosis in China.

## Methods

We translated the smRSq (2011) from English to Chinese with forward and backward translation (Figure S2), to allow for inconsistency detection, and the draft questionnaire was checked for face validity. We enrolled 300 consecutive ischemic stroke patients in the department of neurology, Beijing Chaoyang Hospital, between July and December 2014. We excluded stroke patients who were critically ill on respirators, neurologically unstable, or refused to participate. Also, patients with mild strokes who were released soon after admission, were excluded. We used the World Health Organization definition of stroke [12]. All strokes were confirmed by CT or MRI.

Six raters performed all the ratings within 7 days after admission blinded to the patients’ clinical data and to the other raters’ scores. The six raters consisted of neurology residents between 1 and 3 years in training at our hospital. Each patient was rated by two of the six randomly selected raters. To limit recall bias patients were rated only once on the first day, and to avoid a change in patients functional status, the second rating was done no later than the following day. The first rater in each pair assesses a patient on day one and the second rater on day two, in order to minimize the risk of change in the patient’s condition. If patients could not answer the questionnaire, we interviewed their caregivers. Each rater scored the conventional mRS first, followed by the Chinese version smRSq (2011), and the National Institutes of Health Stroke Scale (NIHSS). Only the second rater scored the Barthel Index (BI), either before or after the NIHSS. The NIHSS indicates stroke severity. The Barthel Index (BI) measures activities of daily living. Each rater estimated their average time to score the smRSq.

Our study was approved by the ethics committee of Beijing Chaoyang Hospital, Capital Medical University. Every patient gave a valid informed consent to participate.

### Statistical analysis

For inter-rater reliability of the conventional mRS and the smRSq (2011), we compared scores between the first and the second rater. We calculated the percent agreement and determined kappa (*κ)* and weighted kappa (*κ*_*w)*_ with 95% confidence intervals (CI). For validity of the smRSq (2011) against the conventional mRS, we compared the smRSq (2011) scores of the first rater to the mRS scores of the second rater. We correlated the Chinese version smRSq (2011) scores with the NIHSS scores by the two raters in each pair. We correlated the Chinese smRSq (2011) scores by the first rater with the BI scores by the second rater (only the second rater scored the BI) using the Spearman’s correlation. We used the Statistical Package for Social Sciences (SPSS) version 16.0 (SPSS Inc., Chicago, IL, USA) for data analysis. We considered kappa values and correlation coefficients > 0.75 as good and > 0.90 as excellent. *P* values less than 0.05 were considered statistically significant.

## Results

Table 1 shows the clinical characteristics and the aggregate scores for each scale of the 300 patients. The average time to score the Chinese smRSq (2011) among all the raters was 70 s.

**Table 1:** Characteristics of the 300 patients in this study

Characteristic	Result
Age, years, mean (SD)	64.9 (12.1)
Men, *n* (%)	220 (73)
Hypertension, *n* (%)	210 (70)
Diabetes mellitus, *n* (%)	88 (29)
Prior stroke, *n* (%)	68 (23)
Coronary artery disease, *n* (%)	55 (18)
Atrial fibrillation, *n* (%)	21 (7)
Current cigarette smoker, *n* (%)	160 (53)
NIHSS score, median (IQR)	4 (1–7)

*SD* standard deviation, *IQR* interquartile range

For the conventional mRS inter-rater reliability, the percent agreement between the raters was 80%, the *κ* = 0.76 (95% *CI*, 0.70–0.81), and the *κ*_*w*_ = 0.93 (95% *CI*, 0.90–0.96).

**Fig. 1:**
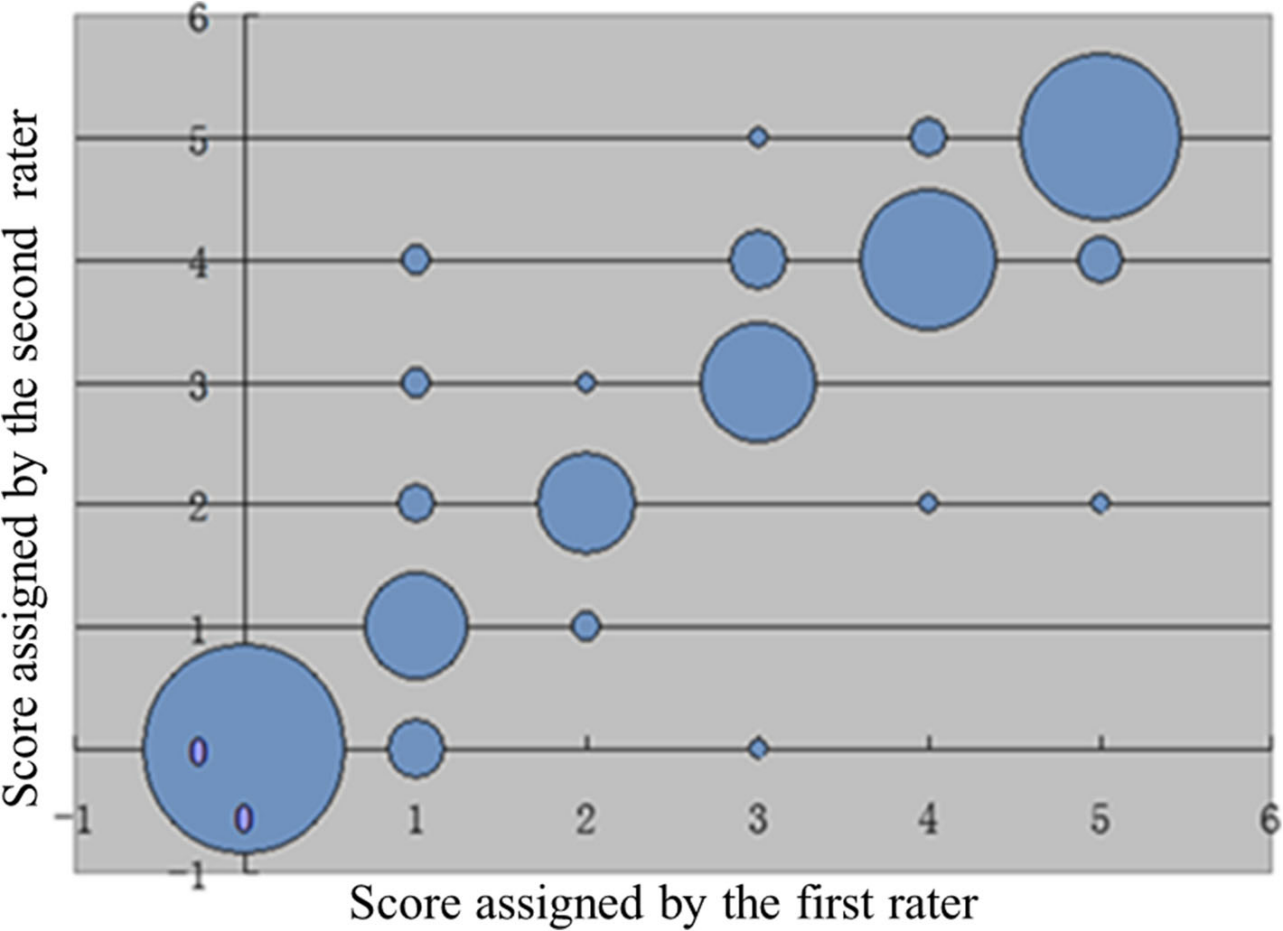
Bubble plot of agreements between the smRSq(2011) scores by the paired raters (diameter of bubbles represents count at each point)

Table 2 shows the cross-tabulation of the smRSq (2011) scores between the paired raters. For inter-rater reliability, the percent agreement between the raters was 87%, the *κ* = 0.84 (95% *CI*, 0.79–0.88), and the *κ*_*w*_ = 0.96 (95% *CI*, 0.95–0.98). Figure 1 illustrates agreement between the smRSq (2011) scores by the paired raters.

**Table 2:** Cross-tabulation of the smRSq(2011) scores between the paired raters

		Second rater						
		0	1	2	3	4	5	Total
First rater	0	**90**	0	0	0	0	0	90
	1	8	**24**	3	2	2	0	39
	2	0	2	**21**	1	0	0	24
	3	1	0	0	**29**	8	1	39
	4	0	0	1	1	**41**	3	46
	5	0	0	1	0	5	**56**	62
Total		99	26	26	33	56	60	300

Comparing the smRSq (2011) scores by the first rater with the conventional mRS scores by the second rater in each pair (Table 3), percent agreement was 55%, *κ* = 0.47 (95% *CI*, 0.40–0.54), and *κ*_*w*_ = 0.91 (95% *CI*, 0.89–0.93). Comparing the smRSq (2011) scores by the second rater with the conventional mRS scores by the first rater produced a similar result (agreement 54%, *κ* = 0.46 (95% *CI*, 0.40–0.52), and *κ*_*w*_ = 0.90 (95% *CI*, 0.88–0.93).

**Table 3:** Cross-tabulation of the smRSq(2011) scores by the first rater and the conventional mRS scores by the second rater

		smRSq(2011) rater1						
		0	1	2	3	4	5	Total
mRS rater2	0	28	3	0	1	0	0	32
	1	62	18	1	0	0	0	81
	2	0	14	22	1	1	1	39
	3	0	2	1	24	1	0	28
	4	0	2	0	13	41	28	84
	5	0	0	0	0	3	33	36
Total		90	39	24	39	46	62	300

In construct validity testing, comparing the smRSq (2011) scores by the first rater with the NIHSS scores by the second rater, the Spearman correlation coefficient was 0.83 (*P* < 0.001). Comparing the smRSq (2011) scores by the second rater with the NIHSS scores by the first rater gave a similar result (Spearman correlation coefficient 0.82). Comparing the smRSq (2011) scores by the first rater with the BI scores by the second rater, the Spearman correlation coefficient was − 0.86 (*P* < 0.001).

## Discussion

Our primary objective in this study was to test the clinimetric properties of a novel Chinese version smRSq (2011). We assessed the inter-rater reliability of the smRSq (2011) and validated it against the conventional mRS interview. We tested the construct validity of the smRSq (2011) against the NIHSS and the BI. We found good to excellent reliability and good validity of the Chinese version smRSq (2011). The Chinese smRSq (2011) questions were understood by the majority of patients and caregivers with little or no explanation, and the scale was easy to administer. Time to score the smRSq was relatively brief (average 70 s).

The inter-rater reliability of the novel Chinese smRSq (2011) was good to excellent (*κ* = 0.84, *κ*_*w*_ = 0.96) and somewhat better than that of the conventional mRS interview (*κ* = 0.76, *κ*_*w*_ = 0.93). Comparing the Chinese smRSq (2011) to the conventional mRS interview showed a lower agreement (*κ* = 0.47), but the disagreements were relatively small as indicated by the excellent weighted kappa of 0.91. Similar inter-rater reliabilities and comparisons to the conventional mRS have been reported using various other aids involving a structured interview [4, 5, 7, 13].

Construct validity testing showed good correlations between the novel Chinese smRSq (2011) and both the NIHSS (0.82–0.83) and the BI (− 0.86). This result is consistent with other validity studies using the conventional mRS [14], the English version smRSq (2011) [15], and our prior study of the Chinese smRSq (2010, 10].

Our results suggest that the novel Chinese smRSq (2011) may be a suitable aid for scoring the mRS in Chinese stroke patients. The advantage of this aid over the conventional mRS interview is simplicity, brevity, and perhaps improved reliability. The significance of this aid is magnified by the relatively large prevalence of stroke in China, and the advantage of acquiring standardized functional outcome measures.

This study has limitations. First, the paired ratings were done on two consecutive days, which may have introduced some recall bias. To limit recall bias we instructed the patients to treat each interview independently of the others. Second, the mRS should ideally be scored after some period of recovery from stroke and in a community setting. Thus, although the scores in our patients likely do not represent their ultimate functional outcome, the paired ratings were done under similar circumstances. Third, we did not test the novel Chinese version smRSq (2011) over the telephone or via telemedicine, and remote outcome assessments are often more practical than in-person assessments.

## Conclusion

In conclusion, this study demonstrates good to excellent reliability and good validity of the novel Chinese smRSq (2011) in scoring the mRS in Chinese stroke patients. The simplicity of the smRSq (2011) aid further augments its usefulness. Additional confirmatory testing of the Chinese smRSq (2011) in out-of-hospital settings, by remote methods, by non-stroke physicians, and by non-physicians is warranted.

## Supplementary information

**Additional file 1: Figure S1.** Slightly revised simplified modified Rankin Scale questionnaire (2011).

**Additional file 2: Figure S2.** The Chinese language smRSq(2011).

## Data Availability

This is a research article and all data generated or analyzed during this study are included in this published article. Data are, however, available from the authors upon reasonable request and with permission.

## Abbreviations

mRS: modified Rankin scale
BI: Barthel index
NIHSS: National Institutes of Health Stroke Scale
smRSq: simplified modified Rankin scale questionnaire
