# Supplementary material for: The reliability and validity of a novel Chinese version simplified modified Rankin scale questionnaire (2011)
Source: BMC Neurol. 2020 Apr 8;20:127. doi: 10.1186/s12883-020-01708-1 (PMC7140377; doi:10.1186/s12883-020-01708-1)

**Figure S2. Slightly revised simplified modified Rankin Scale questionnaire**

(Bruno A, Akinwuntan AE, Lin C, Close B, Davis K, Baute V, et al. Simplified modified rankin scale questionnaire: reproducibility over the telephone and validation with quality of life*.* Stroke. 2011; 42(8): 2276-9.)


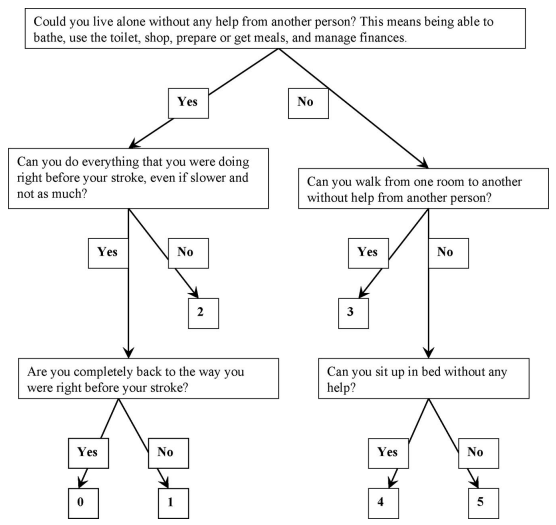

Supplement: Supplementary file 1 — Additional file 1: Figure S1. Slightly revised simplified modified Rankin Scale questionnaire (2011). [file 12883_2020_1708_MOESM1_ESM.docx]
