# Supplementary material for: The reliability and validity of a novel Chinese version simplified modified Rankin scale questionnaire (2011)
Source: BMC Neurol. 2020 Apr 8;20:127. doi: 10.1186/s12883-020-01708-1 (PMC7140377; doi:10.1186/s12883-020-01708-1)
Supplement: Supplementary file 2 — Additional file 2: Figure S2. The Chinese language smRSq(2011). [file 12883_2020_1708_MOESM2_ESM.docx]

**Figure S1. The Chinese language smRSq(2011)**

你能不需要他人帮助独立生活吗？包括洗澡、上厕所、购物、吃饭、管理财务等

你能像卒中以前那样做任何事情吗，尽管动作有点慢且不及以前？

你能不需要他人帮助从一个房间到另一个房间吗？

YES

NO

NO

**2**

YES

你能完全恢复到卒中以前的状态吗？

YES

NO

**0**

**1**

NO

YES

**3**

你能从床上坐起来吗？

YES

NO

**4**

**5**
